# Sexuality of Women after Gynecological Surgeries

**DOI:** 10.3390/healthcare8040393

**Published:** 2020-10-10

**Authors:** Mateusz Kozłowski, Paula Gargulińska, Łukasz Ustianowski, Roksana Lewandowska, Sebastian Kwiatkowski, Aneta Cymbaluk-Płoska

**Affiliations:** 1 Department of Gynecological Surgery and Gynecological Oncology of Adults and Adolescents, Pomeranian Medical University, 70-111 Szczecin, Poland; p.gargulinska@wp.pl (P.G.); l.ustianowski@gmail.com (Ł.U.); roksanna.lew@gmail.com (R.L.); aneta.cymbaluk@gmail.com (A.C.-P.); 2 Department of Obstetrics and Gynecology, Pomeranian Medical University, 70-111 Szczecin, Poland; kwiatkowskiseba@gmail.com

**Keywords:** sexuality, hysterectomy, pain, chemotherapy, quality of life

## Abstract

(1) Background: Disorders of sexual life negatively impact self-esteem and social relationships. This problem affects patients after gynecological surgery. Providing access to specialist sexologist care constitutes an important aspect of support for this patient group. (2) Objective: The aim of the study was to assess the sexual life of women depending on the time since surgery, extent of gynecological surgery and postoperative chemotherapy and/or radiotherapy. (3) Methods: The study included 136 patients from gynecological outpatient clinics in Szczecin, Poland. The women answered questions from a special three-part questionnaire. Participation was anonymous and voluntary. The data obtained in the survey were subject to statistical analysis. (4) Results: Among patients with a sparing of the cervix, most have never or almost never experienced discomfort or pain during intercourse, and believe that the quality of their sex life has not deteriorated after surgery. It was found that cervical removal, despite the existence of other conditions, increases the chance of pain during sexual activity 11 times. We found that the removal of adnexa did not increase the risk of changing sexual activity. In patients who had not undergone postoperative chemo- and/or radiotherapy, sexual activity did not change after surgery, and they never or almost never experienced discomfort or pain during intercourse. On the other hand, it was shown, despite the smaller study group, that patients treated with postoperative chemo- and/or radiotherapy did not initiate sexual intercourse. (5) Conclusion: The more extended the gynecological surgery of the uterus, the greater the limitation of sexual life.

## 1. Introduction

Sexuality is an essential component of human life and is influenced by social attitudes [1]. Problems with sexuality can lead to deterioration of the quality of life and emotional distress. For many patients, sexuality is often a taboo [2]. It should be noted that only some patients bring up the topic of sexuality in a conversation with a doctor. The primary barrier to sexual health discussions is the patient’s discomfort with discussing sexuality [3]. It is necessary to state that there are difficulties in finding a competent person in the field of sexology [4,5]. A lack of interest on the part of doctors regarding the sexual life of their patients is a particular problem in the doctor–patient relationship [4,6,7,8]. Women who are not accustomed to talking about intimate topics often avoid mentioning them. This phenomenon is observed both in oncology patients as well as in those not currently undergoing oncological treatment. This leads to a situation where the lack of questions regarding sexuality leaves patients and partners with many unanswered questions, and feeling that they are unprepared for the sexual side effects of cancer and treatments, lacking in knowledge about the cause and duration of their sexual problems [9]. The apparent lack of interest on the part of the doctor supports their belief that their problem is singular. While healthcare professionals consider support for sexual well-being to be significant, they claim that it is not routinely provided [10]. Moreover, it seems that many patients during cancer therapy trivialize the aspect of sexuality, putting it up against other issues more paramount to be addressed [11]. Undeniably, surgical treatment of the reproductive organ—due to both non-malignant and malignant causes—significantly affects the quality of the sex life. This fact assumes particular weight in patients undergoing oncological treatment, wherein chemical- and/or radiotherapy are often used together in therapeutic management. Both groups of patients are at increased risk of sexual problems—discomfort or pain during intercourse, and changes in sexual activity or sexual interest levels. 

Hysterectomy is one of the most commonly performed gynecological operations in the world [12,13]. The main types of this procedure include excision of the entire uterus (total hysterectomy) or resection of the uterine body (partial hysterectomy). Surgeries can be extended to include the excision of fallopian tubes, ovaries, upper vagina or lymph nodes [14]. Non-malignant lesions of the reproductive organs are the usual indications for uterus excision. The common causes include uterine fibroids, prolapse of pelvic organs, very heavy and painful menstruation, endometriosis, adenomyosis and precancerous lesions of the endometrium and the cervix [15]. The causes of sexual dysfunction after hysterectomy include damage to the branch of the pelvic plexus in different anatomical locations [16,17]. Nevertheless, there are reports in the literature suggesting both the adverse as well as beneficial effects of hysterectomy on sexuality [18,19,20].

The aim of the study was to assess the sexual life of women, depending on the time since surgery, extent of gynecological surgery and postoperative chemotherapy and/or radiotherapy. The authors assumed that the removal of the entire uterus deteriorates the quality of sexual life of the patients more than the removal of the corpus uteri alone. 

## 2. Materials and Methods 

### 2.1. Participants

#### 2.1.1. Participation in the Study

The survey was conducted in women who attended gynecological outpatient clinics in Szczecin (Poland) over the years 2018–2019. Our patients consisted of women from the West Pomeranian, Lubuskie, Greater Poland and Pomeranian provinces. Questionnaires were given to 320 patients. The subjects answered the questions contained in the survey voluntarily and anonymously. The study inclusion criteria were the following: surgery of the reproductive organ during the last year and no re-operations. The following criteria for exclusion from the study were adopted: repeat surgery during the last year, surgery, chemotherapy or radiation therapy for non-gynecological cancers, previous psychological and/or psychiatric treatment, and incomplete filling of the questionnaire. After thorough analysis of the materials, 136 patients were included in the study. 

#### 2.1.2. Characteristics of the Questioned Group

Prior to statistical analysis, the patients were divided into groups based on basic information (A) and surgical information (B). The numerical distribution of the study group according to the place of residence was as follows: country—20 patients; city of 200 thousand inhabitants or more—72; city of 100 to 200 thousand inhabitants—22; city of 20 to 100 thousand inhabitants—12; city of 5 to 20 thousand inhabitants—6; city of 5000 inhabitants or less—4. 

Moreover, subjects were divided according to education, as follows: primary (0), gymnasium (14), vocational (39), secondary (42), higher (41). Among the surveyed patients, 71 gave birth 1–2 times, 44 patients 3 or more times, and 21 patients were nulliparous (Table 1). 

The numerical distribution of patients, taking into account most of the information relating to surgery, is presented in Table 2.

**Figure 1:**
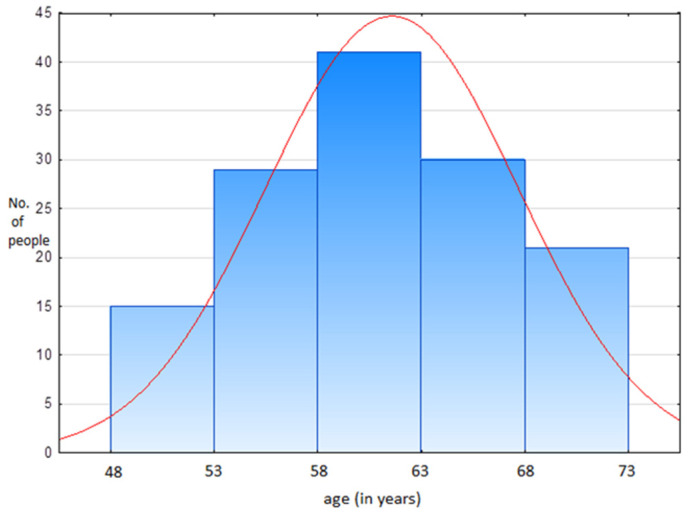
Distribution of patients depending on age (*p* = 0.0244). The red line shows the mean age of the patients.

Most patients (74) underwent surgery 2–5 years ago, 38 underwent surgery 1 year ago or less, 18 patients had a procedure performed 6–10 years ago and 6 patients more than 10 years ago. The patients were also analyzed in terms of age. The age distribution was not normal (*p* = 0.0244). The median age in the study group was 61 years (minimum 48 years, maximum 73 years) (Figure 1).

Laparoscopy was performed in 46 patients (34%) and laparotomy in 90 patients (66%). There were 68 patients (50%) who had undergone salpingo-oophorectomy. Only in 3 cases was there unilateral removal. In this group, 59 patients were taking hormone replacement therapy (HRT), which certainly improved their sex life comfort. Among the patients undergoing oncological treatment, 10 underwent adnexa removal (9 of which were after chemotherapy, 1 after radiotherapy).

Among those surveyed, 89% were not undergoing postoperative chemotherapy and/or radiotherapy, while the remaining group (11%) did receive adjuvant treatment, suggesting that these patients were diagnosed with malignant tumors of the reproductive organs.

### 2.2. Instruments

The study used a specially prepared questionnaire focused on assessing the quality of sex life in women after gynecological surgery. The questionnaires we used in preparing our questionnaire were The Supplemental 24-Item Cervical Cancer Module of the European Organization for Research and Treatment of Cancer Quality of Life Questionnaire (EORTC QLQ-CX24), which is a useful instrument for assessing the quality of life of patients who are treated for cervical cancer [21], and FACT-Cx, which consists of the Functional Assessment of Cancer Therapy (FACT-G) plus a cervix cancer-specific subscale, the Brief Pain Inventory-Short Form (BPI-SF), and a neurotoxicity subscale [22]. The questionnaire was approved by the Pomeranian Medical University in Szczecin. It consisted of three parts: (A) basic information, (B) information regarding surgery, and (C) information on the quality of sex life. It contained 17 semi-open and closed questions. In Part A, patients provided information about the place of residence depending on its population, education (primary, gymnasium, vocational, secondary, or higher), and the number and type of parturitions (force of nature or caesarean section). In Part B of the survey, participants responded to questions regarding how many years ago the surgery was carried out, whether the body of the uterus was removed during the operation, whether the cervix was left and whether the patient underwent chemotherapy and/or radiotherapy after the surgery. In Part C, pertaining to the quality of sex life, subjects answered questions about sexual interest levels, changes to sexual activity after surgery, frequency of experiencing discomfort or pain during intercourse, frequency of attaining vaginal lubrication during sexual activity or intercourse, frequency of orgasm during the erotic stimulation preceding sexual intercourse or intercourse, deterioration of the quality of sex life after surgery, the use of vaginal moisturizers, frequency of vaginal and vulvar infections after surgery, and the development of urinary incontinence after surgery.

### 2.3. Procedure

The surgeries were divided into two categories: the first included surgeries on the uterine body, and the second group consisted of minor gynecological procedures, which included operations on adnexa and on the cervix, and vaginal and vulvar reconstructions.

### 2.4. Data Analysis

The data obtained from the questionnaires were subject to statistical analysis. The assessment of the relationship between qualitative variables was based on the value of χ^2^, which was calculated using contingency tables. Having the statistics for χ^2^, the value of Cramer’s V was determined, which takes up values in the range 〈0,1〉. The higher the value of Cramer’s V, the stronger the relationship between traits. The Cramer’s V was used to determine most of the relationships. A Pearson’s chi-square test was used to analyze the statistical part of the relationship. The details are presented as tables and charts. The analysis of age distribution was performed with the Shapiro–Wilk test. Logistic regression was used to analyze the relationship between (a) cervical removal and the chance of pain during sexual activity, and (b) the removal of adnexa and the risk of changes in sexual activity. The model results are shown as ROC graphs [23,24]. 

### 2.5. Ethical Statement

Resolution number: KB-0012/77/14 of the Bioethics Committee of the Pomeranian University of Medicine in Szczecin. All procedures involving human participants were in accordance with the ethical standards of the institutional and/or national research committee, and with the 1964 Declaration of Helsinki and its later amendments or comparable ethical standards. Written informed consent was provided by the patients and the physician. All patients were encouraged to ask questions about their participation in the study.

## 3. Results

After characterizing the groups of surveyed patients, a statistical analysis was carried out to assess the relationships between group A’s (basic information) or B’s (surgical information) characteristics vs. group C’s (information on the quality of sex life) characteristics.

Comparing the level of sexual interest over time since surgery (*p* = 0.04766), we found that the majority of patients who underwent surgery 2–5 years ago (22.8% of the study group; 42% of those surveyed in the group “2–5 years”) and 1 year or less ago (11.8% of the study group and 42% in the group “1 year or less”) rated their level of sexual interest as moderate. When analyzing the frequency of orgasms achieved during the erotic stimulation preceding sexual intercourse or intercourse over time that elapsed from surgery (V = 0.246), it was noted that the majority of patients (14.7%) underwent surgery 2–5 years ago and most could achieve orgasm.

Among patients after resection of the uterine body (32.4% of subjects), 56.8% reported that their sexual activity decreased after surgery. In contrast, among patients undergoing minor gynecological procedures (67.6% of subjects), 60.9% responded that the activity had not changed (V = 0.232). Moreover, we observed that sexual activity after surgery decreased in 42.6% and increased in 4.4% of patients. Despite the rather poor relationship between individual characteristics (V = 0.299), among the groups who did not have the uterine body removed, 41.3% (27.9% of all patients) reported that the quality of their sex life did not deteriorate after surgery. On the other hand, among patients after uterine body resection, 40.9% (13.2% of the total) believe that the quality of their sex life rather worsened. It should also be noted that weak association has been demonstrated (V = 0.192) between uterine body resection and the development of urinary incontinence in the postoperative period.

**Figure 2:**
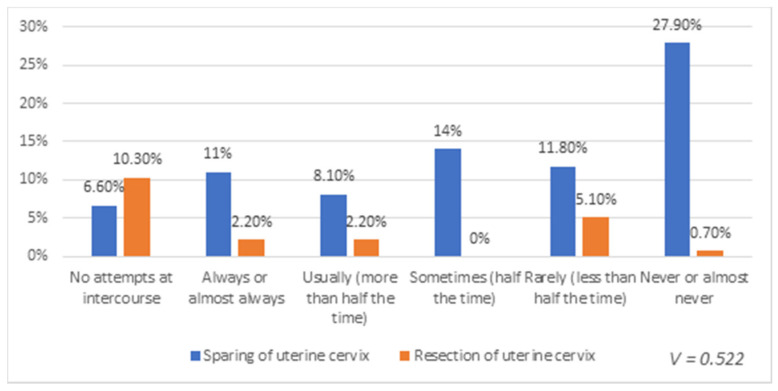
Relationship between the extent of surgery (resection or sparing of the cervix) and the frequency of experiencing discomfort or pain during intercourse.

The relationship between sparing the cervix and quality of sexual life was also analyzed. A strong correlation was demonstrated between the extent of surgery (resection or sparing of the uterine cervix) and the frequency of experiencing discomfort or pain during intercourse, as demonstrated in Figure 2.

**Figure 3:**
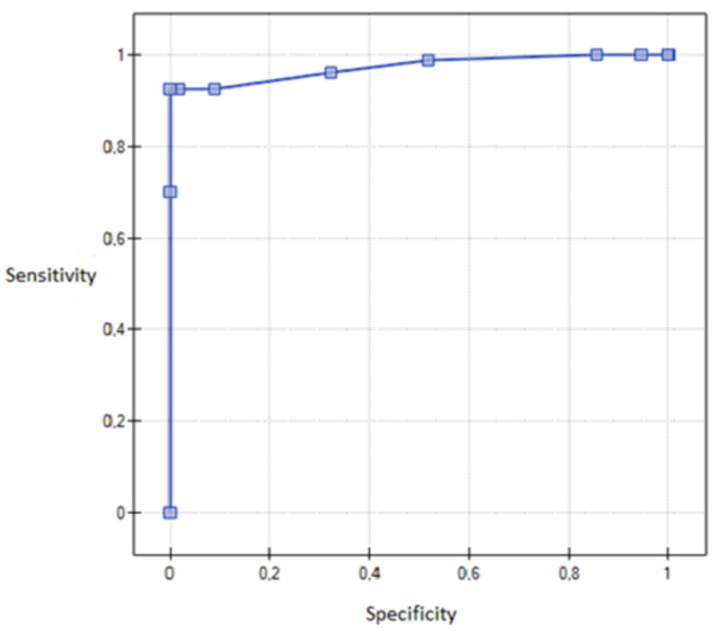
An ROC (Receiver Operating Curve) graph showing logistic regression model—The influence of cervical removal on pain during sexual activity.

It was found that cervical removal, despite the existence of other conditions, increases the chance of pain during sexual activity 11 times (sensitivity (95% CI) = 69.91% (44.61%; 89.02%)) and (specificity (95% CI) = 92.65% (83.22%; 96.67%)), *p* = 0.0062. The model of logistic regression is presented using the ROC curve (Figure 3).

**Figure 4:**
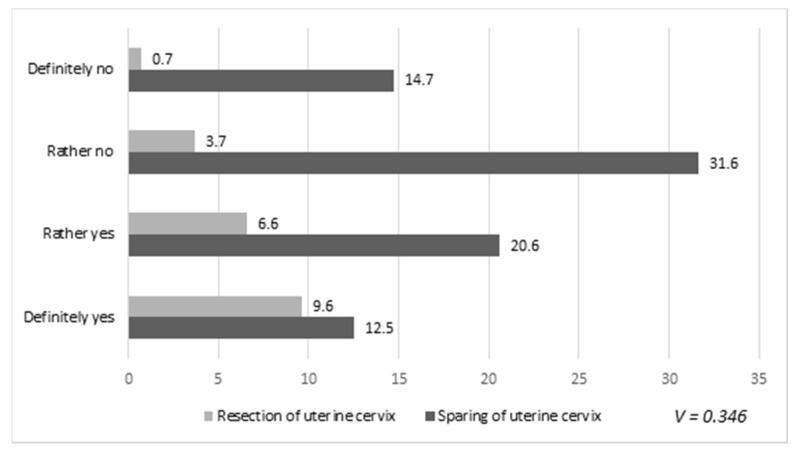
Relationship between the extent of surgery (resection or sparing of the cervix) and deterioration of the quality of women’s sexual lives after surgery. Results are presented as percentages (%).

We also correlated sparing the cervix with deterioration in the quality of sexual life after surgery, as shown in Figure 4. We also noticed a weak relationship between the extent of surgery (resection or sparing of the cervix) and the change in the incidence of vaginal and vulvar infections during the postoperative period.

**Figure 5:**
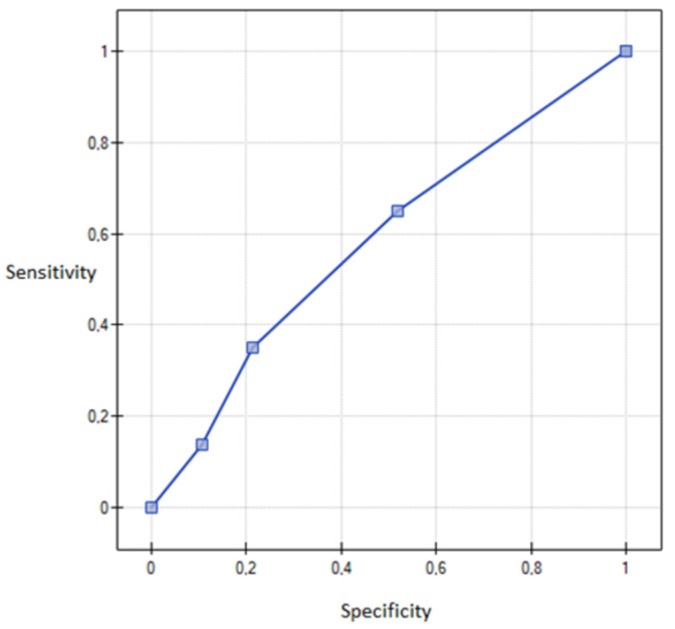
An ROC graph showing the logistic regression model—the influence of salpingo-oophorectomy on changes in sexual activity.

We also focused on salpingo-oophorectomy. It was hypothesized that the lack of adnexa causes a change in the sexual activity of the patients. It should be mentioned that most of the patients took HRT, but the choice of the group was random. We found that the removal of adnexa, despite the existence of other conditions, did not increase the risk of changing sexual activity (*p* = 0.6411) (Figure 5).

**Figure 6:**
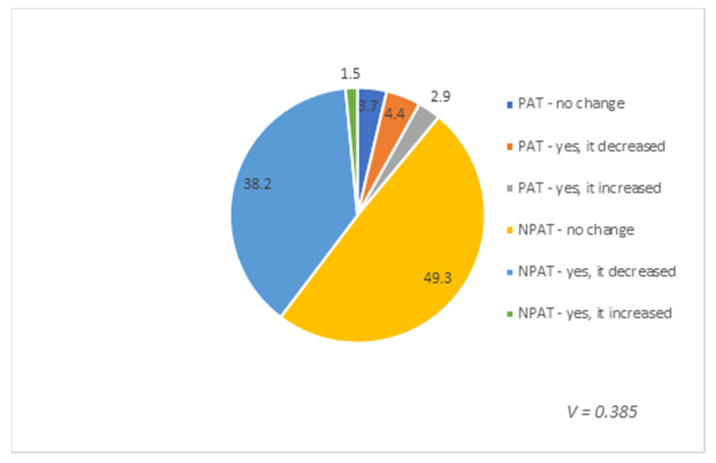
Relationship between postoperative adjuvant chemo- and/or radiotherapy and change in the level of sexual activity after surgery. PAT—postoperative adjuvant treatment. NPAT—no postoperative adjuvant treatment. Results are presented as percentages (%).

The relationship between postoperative chemo- and/or radiotherapy (postoperative adjuvant treatment—PAT) and changes in sexual activity after surgery was also subject to statistical analysis. A strong-to-moderate correlation between traits has been demonstrated. The results are presented in Figure 6.

The relationship between postoperative chemotherapy and/or radiotherapy and the frequency of discomfort or pain during intercourse (V = 0.331) was also assessed; the results are presented in Table 3.

## 4. Discussion

As indicated by our statistics, 58 partial hysterectomies (uterine body resection) and 211 total hysterectomies (resection of uterine body and cervix) were performed at the Department of Gynecological Surgery and Gynecological Oncology of Adults and Adolescents of the Pomeranian Medical University in Szczecin in 2018. 

Our study included 108 patients after amputation of uterine body and 28 patients after excision of the entire uterus. Among patients with sparing of the cervix, most have never or almost never experienced discomfort or pain during intercourse, and do not think that the quality of their sex life has deteriorated after surgery. Among patients with an amputated cervix, most did not attempt intercourse, and believe that the quality of their sex life has definitely deteriorated after surgery. This may be due to the loss of a large part of the sensory weave when removing the neck [16]. An analysis carried out by Wydra [25] on patients after total and supracervical excision of the uterus did not show significant differences with regard to the number of sexual relations, frequency of orgasms, desire for sexual intercourse, pain during intercourse, or vaginal dryness. Similarly, Kuppermann [26] used the Sexual Problem Scale to assess the sexual functioning of women after total and supracervical hysterectomy. They failed to find statistically significant differences between the two groups of patients. Lermann [27] did not observe differences with regard to the incidence of hypoactive sexual drive disorder (HSDD) after hysterectomy using the Brief Profile of Female Sexual Functioning (B-PFSF) questionnaire, regardless of the surgical technique used. Although there are studies demonstrating improvements in sexual functioning in patients after both complete and supracervical hysterectomy [28,29], some of them [30] show that women undergoing partial hysterectomy experience orgasm more often and feel greater sexual pleasure than women after complete hysterectomies. However, the authors themselves point out that the results should be interpreted carefully. The authors pointed out that the fact that most patients knew what type of hysterectomy they had undergone might have been a source of bias. The authors also wrote that due to the strict inclusion criteria, they had a relatively small study group [30]. Moreover, the literature distinguishes between the impacts of various surgical methods on the quality of sexual life. Comparing transvaginal versus transabdominal procedures, no adverse effect on sexual function was reported. To the contrary, improvements in sexual behavior, while reducing the dyspareunia, were noted [31,32]. Ellström [33], on the other hand, comparing abdominal versus laparoscopic hysterectomy suggested that surgical technique has no effect on mental well-being and sexuality after hysterectomy. The literature also contains review articles. Studies from 2000 demonstrated that the quality of life had improved in most women after hysterectomy and that hysterectomy did not adversely affect sexuality [34]. On the other hand, a 2003 study shows the improvement of sexual functioning [35]. Nevertheless, research into the effects of hysterectomy on women’s sexual functioning is inconclusive. Both Farell [34] and Maas [35] indicate that other factors that might affect sexuality should also be taken into account in future investigations.

In our study, the removal of adnexa did not increase the risk of changing sexual activity. In another study, five groups of women were assess, as follows: (i) hysterectomy only; (ii) hysterectomy with BSO (bilateral salpingo-oophorectomy) and no estrogen; (iii) hysterectomy, BSO and estrogen replacement; (iv) hysterectomy, BSO and androgen/estrogen replacement, and (v) non-surgical controls. The authors stated that all surgical groups had more sexual problems than in the control group between 4 months and 5 years post-operation [36]. Teplin et al. described the impact of oophorectomy on the health-related quality of life and sexual functioning in premenopausal women. It was noted that there were no differences in sexual functioning between the groups (hysterectomy and BSO vs. hysterectomy and ovarian conservation) [37]. The 2018 study concluded in turn that abdominal and vaginal hysterectomy with or without bilateral salpingo-oophorectomy for benign causes positively affect female sexuality in general. The authors also noted that premenopausal bilateral oophorectomy may cause more pain during intercourse, as well as decreased libido and orgasm, compared to ovary conservation [38]. Contrary to our results, Tucker et al. have a different opinion on sexuality after oophorectomy. The authors claim that salpingo-oophorectomy can significantly impact on a woman’s sexual well-being. In their study, the most frequently reported symptoms were vaginal dryness and reduced libido [39].

The causes of sexual disorders in patients with malignancies of the reproductive organs include both anatomical changes due to the disease itself, as well as the effects of treatment (chemotherapy, radiotherapy, surgery). The primary aim of treatment is to achieve radical cytoreduction by means of a minimally invasive method, resulting in a higher number of remissions and a better quality of life. As our study shows, in most patients after resection of the uterine body, the sexual activity decreases after surgery. In contrast, in most patients after minor gynecological procedures, the sexual activity is not changed. Among patients after uterine body resection, more than 40% claim that the quality of their sex life has rather worsened. It should be mentioned that not only uterine body resection negatively affects the sexuality of the patients; we found that cervical removal increases the chance of pain during sexual activity. Similar information can be found in the literature. Pieterse et al., while observing patients after radical hysterectomy and pelvic lymphadenectomy due to cervical cancer, found significantly more sexual adverse effects compared to the control group and to patients’ state before surgery. The problems included less lubrication, short and narrow vagina, areas of numbness around the labia, dyspareunia, and lack of sexual satisfaction [40]. A Thai study by Tangjitgamol [41] investigated sexual dysfunction in women after hysterectomy performed due to cervical cancer. The following were evaluated: frequency of intercourse, lust, arousal, lubrication, orgasm, sexual satisfaction and dyspareunia. The following results were obtained: 7.6% did not resume sexual activity after surgery, 92.4% took an average of 4 months after surgery to engage in sex, and dyspareunia increased in 37%. Other sexual functions decreased by 40–60%. Sexual disorders in patients after radical hysterectomy due to cervical cancer were also described by Jensen [42]. However, he noted that most patients who were sexually active before diagnosing the disease returned to activity 12 months after surgery (91%), although with a lower frequency. There are also reports in the literature that present a different position from our research. However, according to Greenwald [43], who studied long-term survivors of cervical cancer, they were involved in satisfactory sexual activity. Among the group of sexually active women (81.1%), 90.9% achieve sexual satisfaction. There are also studies available in the literature that report increased libido after excision of the uterus [19,44].

In our study, 121 women were not subject to postoperative chemo- and/or radiotherapy, suggesting that the reason for surgery was non-malignant, while 15 underwent such an adjuvant treatment. Among patients who had not undergone postoperative chemo- and/or radiotherapy, sexual activity did not change after surgery, and they never or almost never experienced discomfort or pain during intercourse. In turn, among patients treated with postoperative chemo- and/or radiotherapy, despite a smaller group size, we found that most did not try to initiate intercourse. However, in 2.2% of subjects, discomfort or pain during intercourse occurs more than half the time, while in another 2.2% it never or almost never occurs. However, it is uncertain whether the reduced frequency of intercourse is caused by dryness and vaginal shortening in the course of radiotherapy, or the background is psychological. However, this seems to stem from both physical as well as psychological factors [45,46,47]. Frumovitz [48] compared the quality of life and sexual function of patients after radical hysterectomy and lymphadenectomy with those of patients after radiotherapy due to cervical cancer, demonstrating worse results in patients after radiotherapy based on the questionnaires. The negative impact of radiation therapy on sexual performance was also confirmed by Italian studies by Ditto [49], based on the FACT-Cx questionnaire, as well as Danish studies by Jensen [50]. Vaginal stenosis also poses a significant problem for patients after radiotherapy; therefore, various methods of vaginal dilatation are applied. Canadian studies showed vaginal dilation in patients after radiotherapy undergoing a psychoeducational program consisting of “information–motivation–behavioral skills” [51]. On the other hand, according to Miles [52], routine dilatation during or shortly after radiotherapy can be harmful. They found no evidence showing that routine vaginal expansion during or after treatment prevents late complications of radiotherapy and increases the quality of life. Observations were made on the basis of the above-mentioned literature. Thus, the vast majority of studies show that the surgical treatment of gynecological cancers supplemented with chemotherapy and/or radiotherapy negatively affect the sexuality of patients. This is confirmed by Shankar et al.’s latest research. This study shows that a treatment consisting of surgery and radiotherapy in cervical cancer patients causes more sexual dysfunction and dissatisfaction [53]. 

Although our study did not include taking up the subject of sexuality during the conversation with the doctor, we want to point out how important it is for the doctor to approach the subject of sexuality correctly. Therefore, the initiating of a conversation about possible sexual dysfunction by a doctor on one of the first visits would seem like a groundbreaking idea. Moreover, including a sexologist in the medical team could be important for the proper specialized and personal management of oncological patients [54]. This would provide patients with an opportunity to talk to a competent, experienced person, and would aid gynecologists in their daily work. 

The authors state that the objectives have been achieved.

## 5. Conclusions

1. There were no differences in the level of sexual interest between a group of patients who were operated on 1 year ago or less and a group of patients who were operated on 2 to 5 years ago.
2. Minor gynecological procedures do not affect the sexual lives of patients.
3. Discomfort or pain during intercourse occurs more frequently in patients after complete uterine resection.
4. Patients treated oncologically are less likely to engage in sexual activities.

These statements constitute the basis for the following conclusion:

The more extended the gynecological surgery of the uterus, the greater the limitation of subsequent sexual life.

## Figures and Tables

**Table 1:** Relationship between the number of labors and the method of labor (*p* < 0.00001).

Number of Labors	Spontaneous Labor	Cesarean Section	Both	None
0 labor	0	0	0	21
1–2 labor/s	44	22	5	0
≥3 labors	36	0	8	0

**Table 2:** Distribution of patients taking into account surgical data.

Answer of the Respondent (Yes/Not)	Uterine Body Removed	Cervix Intact	Postoperative Chemotherapy and/or Radiotherapy
Yes	44	108	15
Not	92	28	121

**Table 3:** Relationship between the presence of postoperative chemo- and/or radiotherapy and the frequency of experiencing discomfort or pain during intercourse. Results are presented as percentages (%).

Presence of Postoperative Treatment	No Attempt at Intercourse Was Made	Always or Almost Always	Usually (More than Half the Time)	Sometimes (about Half the Time)	Rarely (Less than Half the Time)	Never or Almost Never
Postoperative chemo- and/or radiotherapy	5.1	0.7	2.2	0.7	0	2.2
No postoperative chemo- and/or radiotherapy	11.8	12.5	8.1	13.2	16.9	26.5
